# A Comfort Survey of Timolol Hemihydrate 0.5% Solution Once or Twice Daily *vs* Timolol Maleate in Sorbate

**DOI:** 10.5005/jp-journals-10008-1130

**Published:** 2013-01-15

**Authors:** William C Stewart, Jeffrey C Oehler, Neil T Choplin, Joseph I Markoff, Marlene R Moster, Parul Ichhpujani, Lindsay A Nelson

**Affiliations:** Cofounder and Medical Director, PRN Pharmaceutical Research Network, LLC, Cheyenne Wyoming, USA; Consultant, Northwest Eye Surgeons, Columbus, Ohio, USA; Consultant, Eye Care of San Diego, San Diego, California, USA; Consultant, Philadelphia Eye Associates, Philadelphia, Pennsylvania, USA; Consultant, Ophthalmic Partners of Pennsylvania, Bala-Cynwyd Pennsylvania, USA; Consultant, Glaucoma Facility, Department of Ophthalmology, Government Medical College and Hospital, Chandigarh, India; Consultant, PRN Pharmaceutical Research Network, LLC, Cheyenne Wyoming, USA

**Keywords:** Betimol, Comfort, Glaucoma, Istalol, Timolol hemihydrate, Timolol maleate with potassium sorbate.

## Abstract

**Objective:** To evaluate by survey the comfort upon instillation of timolol hemihydrate compared to timolol maleate with potassium sorbate.

**Design:** A prospective, multicenter, observational, non-interventional study.

**Participants:** One hundred and three patients of open-angle glaucoma or ocular hypertension who were ≥21 years old and were currently prescribed timolol hemihydrate (once or twice daily) or timolol maleate with potassium sorbate once daily as monotherapy or as a part of two-drug therapy.

**Materials and methods:** Study was performed at seven clinical sites in the United States. Patients were surveyed on comfort upon instillation of timolol hemihydrate compared to timolol maleate with potassium sorbate.

**Results:** A difference between timolol hemihydrate and timolol maleate with potassium sorbate for questions 1 (burning/stinging on instillation, p < 0.001) and 4 (tearing on instillation, p = 0.024) was noted. There were no differences between treatment groups for any other question (p > 0.05).

**Conclusion:** This survey suggests that timolol hemihydrate is associated with less stinging/burning and tearing than timolol maleate with potassium sorbate.

**How to cite this article:** Stewart WC, Oehler JC, Choplin NT, Markoff JI, Moster MR, Ichhpujani P, Nelson LA. A Comfort Survey of Timolol Hemihydrate 0.5% Solution Once or Twice Daily *vs* Timolol Maleate in Sorbate. J Current Glau Prac 2013;7(1):11-16.

## INTRODUCTION

Timolol hemihydrate (Betimol^®^, Vistakon Pharmaceuticals, LLC, Jacksonville, FL) was introduced in the 1990s as a low cost alternative to traditional timolol maleate therapy. At that time it was shown that it could be used, like timolol maleate, once daily with equal 24-hour efficacy as monotherapy or added to latanoprost.^1-3^

Several years ago a new timolol maleate preparation became available (Istalol^®^, ISTA Pharmaceuticals, Inc., Irvine, CA) as an alternative once daily prescribed beta-blocker. This preparation contains potassium sorbate which enhances ocular bioavailability, allowing for once daily dosing.^4^ However, timolol maleate with potassium sorbate has been shown to sting upon instillation in approximately 42% of patients.^5^

This study was conducted to evaluate (by survey) the comfort upon instillation of timolol hemihydrate compared to timolol maleate with potassium sorbate.

## MATERIALS AND METHODS

This was a prospective, multicenter, observational (non interventional) study that was performed at seven clinical sites in the United States. The trial was registered with Clinicaltrials.gov, NCT00823043. This study was approved by the Institutional Review Board—Schulman Associates Institutional Review Board, Inc. (SAIRB), 4290 Glendale-Milford Road, Cincinnati, Ohio 45242—and adhered to the tenets of the Declaration of Helsinki. We included patients of open-angle glaucoma or ocular hypertension who were ≥21 years old and were currently prescribed timolol hemihydrate (once or twice daily) or timolol maleate with potassium sorbate once daily as monotherapy or as a part of two-drug therapy. With timolol hemihydrate if one eye was treated QD and the other BID, the QD eye was chosen as the study eye. Similarly, if dosed with two medicines in one eye and one medicine in the other, the lower dosed eye was chosen for the study.

Appropriate consecutive patients who agreed to complete the survey were entered into the trial. After signing informed consent, subjects provided demographic information, medication and ophthalmic history, and then completed a 10-question survey about the comfort and influence on activities of daily living of their current glaucoma medication.

Subjects who met any of the following criteria were not qualified to be enrolled in the trial:

- Inability to understand the trial procedures and thus inability to give informed consent.
- Inability to understand, read or write English.
- Best corrected visual acuity of 20/200 or worse in each eye.
- Current moderate to severe infectious or inflammatory condition of the eye or eyelids including: Blepharitis, conjunctivitis, keratitis or uveitis.
- Current moderate to severe dry eye syndrome.
- Current chronic use of ocular corticosteroids.
- Ocular surgery or intraocular laser surgery to either eye in the prior 3 months.
- Treated with three or more glaucoma medicines in both eyes.

All data collected in the study were from the survey and no patient identifying information was kept. No randomization of treatment assignments was conducted but in general, investigational sites balanced the surveys collected between both groups of treated patients. Investigators were instructed regarding the protocol and related materials during a Site Initiation Teleconference conducted by the Clinical Research Associate (CRA) who had been trained by the CRO. Investigational sites were instructed to review each subject's survey to ensure all questions had been answered before collecting from the subject. The same CRA conducted a study close-out teleconference.

No safety and efficacy variables were measured in the study. The endpoints evaluated were each subject's response to survey questions about their current timolol treatment.

Patient survey shows the survey questionnaire. A 5 point scoring (0 to 4) was done for each of the questions. All questions were analyzed between timolol hemihydrate *vs* timolol maleate with potassium sorbate with a Student's t-test except question 9 which was evaluated with a Chi-square test.^6^ All analyses were two-tailed, with a p-value of 0.05 used to declare statistical significance.

**Table:** 

**Patient survey**	
1. Does your beta-blocker drops burn or sting when you put them in your eyes?	
a. Never	
b. Rarely	
c. Sometimes	
d. Frequently	
e. Always	
2. I would rate my overall tolerability with my beta-blocker drops as:	
a. Excellent	
b. Very good	
c. Satisfactory	
d. Poor	
e. Terrible	
3. I would rank the overall comfort with my beta-blocker eye drops as:	
a. Excellent	
b. Very good	
c. Satisfactory	
d. Poor	
e. Terrible	
4. After you instill them in your eyes do your beta-blocker drops cause tearing?	
a. Never	
b. Rarely	
c. Sometimes	
d. Frequently	
e. Always	
5. After you instill them in your eyes, do your beta-blocker drops cause light to hurt your eyes?	
a. Never	
b. Rarely	
c. Sometimes	
d. Frequently	
e. Always	
6. After you instill them in your eyes, do your beta-blocker drops blur your vision?	
a. Never	
b. Rarely	
c. Sometimes	
d. Frequently	
e. Always	
7. My beta-blocker eye drops cause me:	
a. No disruption of my daily activities (go to question 10)	
b. To pause briefly, but do not disrupt my daily activities	
c. To pause and they limit slightly my daily activities	
d. To pause and they limit significantly my daily activities	
e. To pause and they cause me to stop my daily acitivities	
8. If your beta-blocker drops disrupt your daily activities, for how long?	
a. ≤1 minute	
b. ≤10 minutes	
c. ≤60 minutes	
d. ≤12 hours	
e. ≤24 hours	
f. ≥24 hours	
9. If your beta-blocker drops disrupt your daily activities, which ones specifically (you may mark more than one)?	
a. Reading or other close manual work	
b. Computer work	
c. Managing stairs	
d. Crossing traffic lanes	
e. Food preparation	
f. Other (please specify)	
Answer only, if you are using another glaucoma eyedrop as well as your beta-blocker drops.	
10. I would rate my overall comfort with my beta-blocker eye drops compared to my other glaucoma medicines as:	
a. Much better	
b. Somewhat better	
c. No difference	
d. Somewhat worse	
e. Much worse	

## RESULTS

The study included 103 patients from seven centers. The demographic and baseline data is elucidated in Table 1. The mean age was 70.7 ± 11.4 years. No difference existed between groups for any demographic parameter (p > 0.05) except for age for which timolol maleate with potassium sorbate patients were older (p = 0.04).

The results for all patients for the survey questions are shown in Table 2. There was a difference between timolol hemihydrate and timolol maleate with potassium sorbate for questions 1 (burning/stinging on instillation, p < 0.001) and 4 (tearing on instillation, p = 0.024). However, there were no differences between treatment groups for any other question (p > 0.05). Also, there was little effect on activities of daily living. Question 9 noted that nine patients (14%) treated with timolol hemihydrate and 6 (15%) with timolol maleate with potassium sorbate were limited to at least one daily activity of living that they ascribed to the beta-blocker therapy (p = 0.41).

The same statistical results as with the complete patient population also held with subanalyses of timolol hemihydrate once daily or twice daily *vs* timolol maleate with potassium sorbate, or mono- or adjunctive treatment with timolol hemihydrate *vs* timolol maleate with potassium sorbate. In contrast, no differences were observed for any question between timolol hemihydrate once daily *vs* twice daily treatment (p > 0.05) (Tables 3 to 7).

**Table 1:** **Table 1:** Demographic and baseline data of enrolled subjects

		*N*		*Percentage (%)*	
**A**					
*Gender*					
Male		35		34	
Female		68		66	
Total		103			
*Race*					
Caucasian		73		71	
African American		18		17	
Hispanic		5		5	
Asian		3		3	
Other		4		4	
Total		103			
**B**					
*Significant ophthalmic history*					
Primary open angle glaucoma		93		90	
Ocular hypertension		18		17.5	
Cataracts		13		12.6	
Pseudophakia		3		2.9	
Central retinal vein occlusion		1		1.0	
Diabetic retinopathy		1		1.0	
Macular degeneration		4		3.9	
Significant dry eyes		1		1.0	
Pan retinal photocoagulation		2		1.9	
Selective laser trabeculoplasty		1		1.0	
*Ophthalmic medication history*					
Betimol (timolol hemihydrate)		67		65.0	
Istalol (timolol maleate in sorbate)		39		37.9	
Travatan Z (travoprost BAC free)		17		16.5	
Xalatan (latanoprost)		12		11.7	
Lumigan (bimatoprost)		10		9.7	
Diamox (acetazolamide)		1		1.0	
Azopt (brinzolamide)		1		1.0	
*Therapy type*					
Monotherapy		63		61.2	
Adjunctive therapy		40		38.8	

**Table 2:** **Table 2:** Overview of outcome of survey questionnaire

*Question*		*Timolol hemihydrate*						*Timolol in sorbate*				*p-value*	
		*N*		*Mean score*				*N*		*Mean score*			
1. Burning/stinging?		64		1.1 ± 1.1				39		2.4 ± 1.4		<0.001	
2. Overall tolerability?		64		0.8 ± 0.8				39		1.0 ± 0.9		0.28	
3. Overall comfort?		64		0.8 ± 0.8				39		1.1 ± 1.0		0.14	
4. Tearing?		64		0.9 ± 1.0				39		1.6 ± 1.6		0.024	
5. Light sensitivity?		63		0.5 ± 0.7				38		0.4 ± 0.9		0.75	
6. Blurred vision?		64		0.7 ± 1.0				39		0.6 ± 1.0		0.69	
7. Disruption of daily activities?		63		0.1 ± 0.3				39		0.2 ± 0.5		0.53	
8. How long was disruption?		11		0.2 ± 0.4				7		0.1 ± 0.4		0.84	
10. Overall comfort *vs* other glaucoma medicines?		39		1.5 ± 0.9				26		1.6 ± 1.1		0.94	
Question 9													
*response*		*Timolol hemihydrate*						*Timolol maleate in sorbate*				*Total*	
1				5						5		10	
2				2						0		2	
6				2						1		3	
Total				9						6		15	
p-value**										0.41			

*2-tailed p-value, t-test; **Chi-square test

## DISCUSSION

Timolol maleate formulation combined with sorbate has the advantage of better penetration due to enhanced lipophilicity, but maleate is a salt of an organic acid that has several types of cellular effects. It reduces glutathione, a free radical scavenger, in both epithelial and endothelial cells and in corneal endothelial cells specifically.^7^ Timolol hemihydrate eliminates the maleate buffer and simplifies the purification step for the R-enantiomer.

Betimol^®^ is an isotonic, microbiologically preserved aqueous solution which has monosodium and disodium phosphate dihydrate to adjust its pH (6.5-7.5) and an osmolality of 260 to 320 mOsmol/kg. Timolol maleate too has a pH of 6.5 to 7.5 and an osmolality of 275 to 330 mOsmol/kg. Despite similar pH and osmolaity, stinging is more marked with the latter.

**Table 3:** **Table 3:** Survey question results timolol hemihydrate BID *vs* QD

*Question*		*Timolol hemihydrate BID*						*Timolol hemihydrate QD*					
		*N*		*Mean score ± SD*				*N*		*Mean score ± SD*		p-value*	
1		31		1.2 ± 1.2				32		1.1 ± 0.9		0.63	
2		31		0.8 ± 0.9				32		0.7 ± 0.7		0.54	
3		31		0.9 ± 0.9				32		0.8 ± 0.7		0.54	
4		31		1.0 ± 1.1				32		0.8 ± 0.7		0.28	
5		31		0.6 ± 0.8				31		0.3 ± 0.7		0.12	
6		31		0.7 ± 1.0				32		0.7 ± 1.0		0.82	
7		31		0.1 ± 0.3				31		0.1 ± 0.3		1.00	
8		6		0.2 ± 0.4				5		0.2 ± 0.4		0.90	
10		16		1.3 ± 0.9				22		1.7 ± 0.8		0.10	
Question 9													
*response*		*Timolol hemihydrate BID*						*Timolol hemihydrate QD*				*Total*	
1				3						2		5	
2				1						1		2	
6				0						2		2	
Total				4						5		9	
p-value**								0.34					

*2-tailed p-value, t-test; **Chi-square test

**Table 4:** **Table 4:** Survey question results timolol hemihydrate BID *vs* timolol maleate in sorbate

*Question*		*Timolol hemihydrate BID*						*Timolol maleate in sorbate*					
		*N*		*Mean score ± SD*				*N*		*Mean score ± SD*		p-value*	
1		31		1.2 ± 1.2				39		2.4 ± 1.4		<0.001	
2		31		0.8 ± 0.9				39		1.0 ± 0.9		0.53	
3		31		0.9 ± 0.9				39		1.1 ± 1.0		0.32	
4		31		1.0 ± 1.1				39		1.6 ± 1.6		0.07	
5		31		0.6 ± 0.8				38		0.4 ± 0.9		0.35	
6		31		0.7 ± 1.0				39		0.6 ± 1.0		0.66	
7		31		0.1 ± 0.3				39		0.2 ± 0.5		0.60	
8		6		0.2 ± 0.4				7		0.1 ± 0.4		0.92	
10		16		1.3 ± 0.9				26		1.6 ± 1.1		0.33	
*Question 9*													
*response*		*Timolol hemihydrate BID*						*Timolol maleate in sorbate*				*Total*	
1				3						5		8	
2				1						0		1	
6				0						1		1	
Total				4						6		10	
p-value**						0.33							

*2-tailed p-value, t-test; **Chi-square test

**Table 5:** **Table 5:** Survey question results timolol hemihydrate QD *vs* timolol maleate in sorbate

*Question*		*Timolol hemihydrate QD*						*Timolol maleate in sorbate*				p-value*	
		*N*		*Mean score ± SD*				*N*		*Mean score ± SD*			
1		32		1.1 ± 0.9				39		2.4 ± 1.4		<0.001	
2		32		0.7 ± 0.7				39		1.0 ± 0.9		0.19	
3		32		0.8 ± 0.7				39		1.1 ± 1.0		0.09	
4		32		0.8 ± 0.7				39		1.6 ± 1.6		0.005	
5		31		0.3 ± 0.7				38		0.4 ± 0.9		0.60	
6		32		0.7 ± 1.0				39		0.6 ± 1.0		0.84	
7		31		0.1 ± 0.3				39		0.2 ± 0.5		0.60	
8		5		0.2 ± 0.4				7		0.1 ± 0.4		0.82	
10		22		1.7 ± 0.8				26		1.6 ± 1.1		0.53	
Question 9													
*response*		*Timolol hemihydrate QD*						*Timolol maleate in sorbate*				*Total*	
1				2						5		7	
2				1						0		1	
6				2						1		3	
Total				5						6		11	
p-value**						0.28							

*2-tailed p-value, t-test; **Chi-square test

The reason for the differences between these medications in comfort were not clear by the results of this survey. Past research has also not been able to elucidate if the irritation from timolol maleate results from the active ingredient or the maleate. The lack of effect on activities of daily living may have been related to the resolution of any post-instillation stinging/burning or tearing before these daily activities needed to be performed.

Sonty S et al compared the symptoms and tolerability of once daily timolol hemihydrate 0.5%, timolol in sorbate 0.5%, and timolol gel-forming solution 0.5% in patients with glaucoma and/or ocular hypertension and found that the short-term (3-day) administration of timolol in sorbate was associated with more stinging or burning compared with timolol hemihydrate and timolol gel-forming solution.^8^

**Table 6:** **Table 6:** Survey question results adjunctive therapy timolol hemihydrate *vs* timolol maleate in sorbate

*Question*		*Timolol hemihydrate adjunctive*						*Timolol maleate in sorbate adjunctive*				p-value*	
		*N*		*Mean score ± SD*				*N*		*Mean score ± SD*			
1		24		1.0 ± 0.9				16		2.6 ± 1.4		<0.001	
2		24		0.6 ± 0.6				16		1.0 ± 0.9		0.08	
3		24		0.8 ± 0.7				16		1.2 ± 1.0		0.13	
4		24		0.5 ± 0.9				16		1.8 ± 1.7		0.01	
5		24		0.3 ± 0.6				16		0.3 ± 0.6		0.67	
6		24		0.6 ± 0.9				16		0.9 ± 1.2		0.38	
7		24		0.1 ± 0.3				16		0.2 ± 0.5		0.69	
8		4		0.3 ± 0.5				4		0.0 ± 0.0		0.39	
10		23		1.7 ± 0.6				15		1.5 ± 1.1		0.55	
Question 9													
*response*		*Timolol hemihydrate adjunctive*						*Timolol maleate in sorbate adjunctive*				*Total*	
1				2						2		4	
2				1						0		1	
6				1						1		2	
Total				4						3		7	
p-value**						0.65							

*2-tailed p-value, t-test; **Chi-square test

**Table 7:** **Table 7:** Survey question results monotherapy timolol hemihydrate *vs* timolol maleate in sorbate

*Question*		*Timolol hemihydrate monotherapy*						*Timolol maleate in sorbate monotherapy*					
		*N*		*Mean score ± SD*				*N*		*Mean score ± SD*		p-value*	
1		40		1.3 ± 1.2				23		2.4 ± 1.4		0.003	
2		40		0.9 ± 0.8				23		1.0 ± 1.0		0.74	
3		40		0.9 ± 0.8				23		1.1 ± 1.0		0.47	
4		40		1.2 ± 0.9				23		1.4 ± 1.5		0.46	
5		39		0.6 ± 0.8				22		0.5 ± 1.1		0.94	
6		40		0.8 ± 1.0				23		0.4 ± 0.7		0.12	
7		39		0.1 ± 0.3				23		0.2 ± 0.4		0.64	
8		7		0.1 ± 0.4				3		0.3 ± 0.6		0.64	
10		16		1.3 ± 1.1				11		1.6 ± 1.0		0.44	
Question 9 *response*		*Timolol hemihydrate monotherapy*						*Timolol maleate in sorbate monotherapy*				*Total*	
1				3						3		6	
2				1						0		1	
6				1						0		1	
Total				5						3		8	
p-value**								0.45					

*2-tailed p-value, t-test; **Chi-square test

Our survey did not evaluate symptoms or signs after instillation or adherence in a randomized prospective clinical trial. Further research is needed to analyze anterior segment safety between these two beta-blockers as well as to describe the mechanism of stinging associated with timolol maleate with potassium sorbate.

## SUMMARY

This survey demonstrates that topical timolol hemihydrate is associated with less stinging/burning and tearing than timolol maleate with potassium sorbate.
